# Transcriptome Comparison between Fetal and Adult Mouse Livers: Implications for Circadian Clock Mechanisms

**DOI:** 10.1371/journal.pone.0031292

**Published:** 2012-02-21

**Authors:** Chengwei Li, Shuang Yu, Xiaoling Zhong, Jianguo Wu, Xiaodong Li

**Affiliations:** National Key Laboratory of Virology, College of Life Sciences, Wuhan University, Wuhan, Hubei Province, People's Republic of China; Vanderbilt University, United States of America

## Abstract

Microarray transcriptome analyses of fetal mouse liver did not detect circadian expression rhythms of clock genes or clock-controlled genes, although some rhythmic transcripts that were likely not driven by endogenous cellular clocks were identified. This finding reveals a key distinction between the circadian oscillators in fetal and adult mouse livers. Thus, in this study, the transcriptomes of fetal and adult livers were systematically compared to identify differences in the gene expression profiles between these two developmental stages. Approximately 1000 transcripts were differentially enriched between the fetal and adult livers. These transcripts represent genes with cellular functions characteristic of distinct developmental stages. Clock genes were also differentially expressed between the fetal and adult livers. Developmental differences in liver gene expression might have contributed to the differences in oscillation status and functional states of the cellular circadian clock between fetal and adult livers.

## Introduction

Circadian oscillations are generated by transcription-translation feedback loops formed by clock genes [1], [2]. Most cells have endogenous circadian clocks [3], [4], [5]. However, some cell types appear to lack molecular rhythmicity. Embryonic and fetal tissues develop under intrinsic developmental programs. Recently, mouse embryonic stem (ES) cells were reported not to possess oscillating circadian clocks at the individual cell level [6]. Circadian oscillation gradually appeared during *in vitro* differentiation of ES cells. When differentiated cells were reprogrammed to induced pluripotent stem (iPS) cells, cellular circadian oscillations disappeared again [6]. Mice bearing mutations in key clock genes are viable, revealing that circadian oscillations are not essential to embryogenesis and development. Suppression of circadian rhythms during early development may actually be necessary for proper development. The oscillatory status of the cellular circadian clocks during embryonic and fetal development remains to be fully elucidated [7]. In particular, studies on fetal liver during late gestation in mice or rats could not detect rhythmic expression of several clock genes at the tissue level [8], [9].

We recently performed microarray analyses on fetal liver tissues (results presented in the accompanying paper). We did not detect circadian rhythms in transcript abundance for many of the clock genes and clock-controlled genes that are rhythmically expressed in adult liver (results presented in the accompanying paper). A set of robustly rhythmic transcripts were present in the fetal liver, which may have been regulated by maternal cues. These results indicate that the regulation of gene expression rhythms probably differ between the fetal and adult liver.

Liver metabolic functions undergo adaptive changes during ontogeny [10]. It is expected that regulation of the fetal liver transcriptome would also differ from those in the adult organ. Characterization of the general differences between fetal and adult livers could help to put their differential clock oscillation status into the general context of liver development and function. To this end, we systematically compared the transcriptomes of fetal and adult mouse livers. *In silico* comparisons of our fetal liver microarray data with those for adult mouse liver previously deposited in the public database led to identification of approximately 1000 differentially expressed transcripts, including some clock genes. The implications of those developmental differences in liver gene expression for the differences in circadian clock oscillation status and functional state are discussed.

## Materials and Methods

This study was carried out in accordance with the recommendations in the Guide for the Care and Use of Laboratory Animals of the National Institutes of Health. The study protocol was approved by the Committee on Experimental Animals of the Science and Technology Department of Hubei Province, China (Permit Number: SYXK 2006-0037). Tissue collection, RNA extraction, microarray hybridization and scanning, and general data analyses were carried out as described in the accompanying paper.

### RNA analysis

Based on previous results, we expected a major difference between time-points in gene expression level, particularly in adult liver. As a result, no single time point adequately represents the gene expression profile of a tissue, and this would confound a comparison between fetal and adult liver. To nullify potential circadian differences in gene expression, we created pools of RNA that represented all time-points for fetal (gestation day 18–19) and adult (male, about 3 months old) liver. To pool RNA samples, 0.5 µg of RNA from each time point (embryonic liver tissues: 12 time points for each data series; adult liver tissues: 6 time points) were mixed, 1 µg aliquots were reverse transcribed. For semi-quantitative RT-PCR, equal efficiencies of different reverse transcriptions were validated by PCR analyses of *Actb* for 20 or 23 cycles. Semi-quantitative RT-PCRs on other transcripts were performed for 23, 25, 27 or 30 cycles according to transcript abundance and to facilitate clear contrast. Results presented are representative of duplicate or triplicate repeats. Semi-quantitative RT-PCR products were separated by agarose gel electrophoresis and visualized by ethidium bromide staining and UV transillumination. PCR products were also cloned and sequence verified.

Real-time RT-PCR was performed using the SYBR Green I dye and the difference in Ct values between fetal and adult tissues were compared. For comparisons between fetal and adult liver tissues, multiple reverse transcriptions (six at a time) were performed on aliquots of the same pooled RNA samples. Products were pooled to average out potential variations in efficiencies of different reverse transcription reactions. Real-time RT-PCR analyses were then performed in triplicates. Reverse transcriptions and real-time RT-PCR were repeated independently three times. SYBR Green I signals were read at both 78°C and 85°C, allowing selection of temperatures to eliminate signals from non-specific products if present. Controls were performed on RNA templates without reverse transcriptions. The GenBank accession numbers for the genes targeted by each of the PCR primers used in our study are listed in Table S5.

### *In silico* comparisons between expression values from different data series and across array platforms

The GEO repository accession number for the two series of fetal mouse liver microarray data presented in this study is GSE28622, which contains 24 sample files. We refer to GSM709400-GSM709411 as our series 1 and GSM709521-709532 as our series 2 throughout the text. The GSE11923 Gene Expression Omnibus dataset [11] was used as the reference transcriptome for adult liver (www.ncbi.nlm.nih.gov/projects/geo/query/acc.cgi?acc=GSE11923), which had been generated from the Affymetrix Mouse genome 430 2.0 microarray chip, sharing 22626 probe sets (including several unmapped ones) in common with the Affymetrix Mouse genome 430A 2.0 chip we used in this study. Data series were separately processed using the Robust Multi-Array Average with GC-content background correction (GC-RMA) probe summarization algorithm to obtain normalized expression values. To account for differences in microarray hybridizations and image acquisitions between the data series, average expression values (12 time points at 4 hrs resolution for each of our fetal liver data series, or 48 time points at 1 hr resolution for GSE11923) were calculated for the 22626 common probe sets individually (probe set average). The average values for the 22626 probe set average values were also calculated for the fetal and adult liver tissues respectively (overall average). Since those probe sets represented the majority of transcripts coded in the mouse genome, we assumed that they should have about equal overall average values. Thus, the overall average values for fetal and adult tissues were scaled to the same level, and the scaling factors were then used to scale individual probe set average values. Probe set average values for fetal and adult livers after scaling were compared in a pairwise manner to determine fold differences *in silico*. The *in silico* fold differences are merely reflections of relative abundance, and are not numerically accurate values. Some of the predicted differences were chosen for verification by semi-quantitative RT-PCR. For about 60 different transcripts we studied, the *in silico* differences were all confirmed with just 1 exception. Thus it was estimated that at least 95% of the *in silico* predictions were reliable. *In silico* comparisons were also made between our two data series on fetal livers, which had been hybridized and scanned separately.

### Comparison of phase distribution of rhythmic transcripts in fetal and adult livers

To facilitate phase comparison between fetal and adult livers, JTK_CYCLE was performed using a fixed 24 hours period. Phases (circadian time of expression peak) of rhytmic transcripts were determined using the Lag value given by JTK_CYCLE, and the starting time of each data series (GSE11923 started at CT18; GSE28622 started at CT2) was taken into account. Phase differences were determined. Phases were also converted to angular data and circular-circular correlation analyses were performed using Oriana (v4.00) between rhythmic transcripts common to fetal and adult livers.

## Results

### Differential enrichments of transcripts between fetal and adult mouse livers

**Figure 1:**
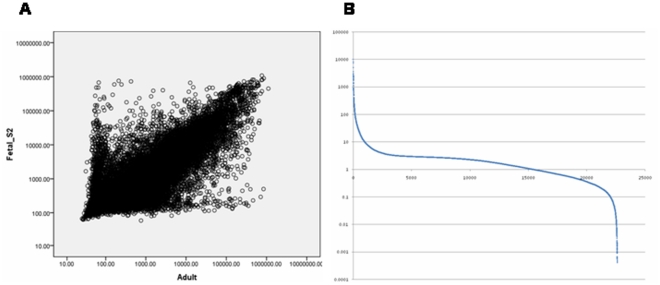
Comparisons between fetal and adult liver transcriptomes. (**A**) Scatterplot of normalized and scaled average expression values in fetal (y-axis, series 2) and adult (x-axis) livers. Pairwise values for 22626 probe sets were plotted. *r* = 0.67, *P* = <0.01. (**B**) Fold differences in normalized and scaled average expression values between fetal (series 2) and adult (GSE11923) livers for 22626 probe sets. Ratios (fetal: adult) were plotted against their ranks.

**Figure 2:**
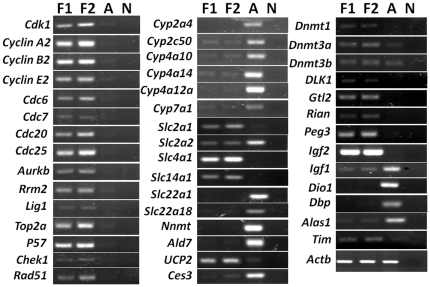
Semi-quantitative RT-PCR analyses on selected transcripts differentially expressed between fetal and adult livers. Equal amounts of starting RNA were reverse transcribed and subject to semi-quantitative PCR. PCR cycles were adjusted depending on transcripts abundance. Fetal (series 1 and 2: F1 and F2) and adult liver (A) PCR results were compared by agarose gel electrophoresis. Negative controls (N) were performed by using no RT templates. All PCR products were sequence confirmed.

At late gestation, fetal liver undergoes a transition from proliferation to functional differentiation [12]. Liver metabolism has been suggested to undergo adaptive changes at several developmental stages, including around birth and after weaning, when dramatic alterations in nutrient source and composition occur [10], [13]. Thus, due to differences in developmental state, differences in gene expression are to be expected between fetal and adult livers. We performed *in silico* comparisons between relative expression levels in the fetal and adult livers for all probe sets represented on the microarray chip we used (Table S1). While our two series of fetal microarray data showed an overall correlation of 0.98 (Figure S1), the fetal and adult liver transcriptomes were also correlated (Figures 1A and S1A), indicating a major portion of the transcriptome did not change dramatically during ontogeny. However, about 1000 transcripts exhibited significant differences (enrichment threshold: > = 10-fold *in silico*) in relative expression levels between fetal and adult livers (Figures 1B and S1B). The 10-fold enrichment criterion used in this study was a convenient and conservative cutoff, and corresponds to the maximum difference observed for pairwise comparisons between our two series of fetal liver data (Figure S2A and S2B) and also for comparisons of daily averages between adult mouse data over two days (Table S2). The Database for Annotation, Visualization and Integrated Discovery (DAVID) was used to analyze fetal liver enriched probe sets, and revealed significant annotation clusters for mitosis, cell cycle control, DNA replication and DNA metabolism (Table S3). For example, *Cdk1*, *Cyclins A2*, *B1*, *B2*, *D2*, *D3*, *E1*, *E2* and other genes involved cell cycle progression (*Aurka* (aurora kinase A), *Aurkb*, *Cdc6*, *Cdc7*, *Cdc20* and *Cdc25b*), and transcripts involved in DNA synthesis and replication (*Rrm2* (ribonucleotide reductase M2), *Lig1* (ligase 1) and *Top2a* (DNA topoisomerase II alpha)) were enriched in the fetal liver. Transcripts related to DNA damage repair (*Exo1* and *Rad51*) and inhibitory genes in cell cycle progression (*Chek1*, *Chek2* and *p57*) were also enriched in the fetal liver. DNA methyltransferases *Dnmt1* and *Dnmt3a* were enriched in the fetal liver. *Dnmt3b* was also expressed at higher relative levels in the fetal liver (but by less than 10-fold *in silico*). On the other hand, nearly 30 cytochrome P450 members (*CYPs*), including *Cyp2a4/5*, *Cyp4a10*/*Cyp4a31*, *Cyp4a12a* and *Cyp7a1* were enriched in the adult liver. Expression of members of the solute carrier family differed by developmental state. For example, *Slc2a1*/*GluT1*, *Slc4a1* and *Slc14a1* were enriched in the fetal liver, while *Slc22a1* and *Slc22a18* (a paternally imprinted gene) were enriched in the adult liver. *Slc2a2/GluT2* was several-fold higher in the adult liver. Many other members of solute carrier family were expressed similarly *in silico*. We noticed that imprinted genes such as *Dlk1*, *Gtl2/Meg3*, *H19*, *Igf2*, *Peg3* and *Rian* [14], were enriched in the fetal liver. *Igf2r* was also expressed at a higher level in the fetal liver, but did not achieve 10-fold enrichment *in silico*. Related but not imprinted genes, such as *Dio1* and *Igf1*, were enriched in the adult liver. We performed semi-quantitative RT-PCR on selected transcripts of interest (∼5% of the total number of transcripts differentially enriched between fetal and adult livers) and confirmed the *in silico* analyses (Figure 2).

### Differential expressions of clock genes and some clock-controlled transcripts between fetal and adult livers

**Figure 3:**
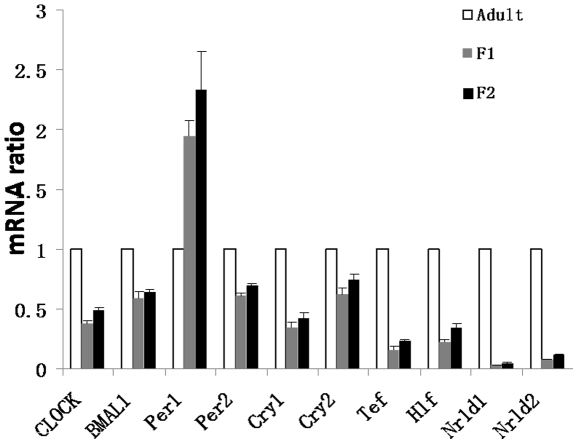
Differential expression of clock genes between fetal and adult livers. Relative expression levels of selected clock genes, as detected by real-time RT-PCR results analyses on. Delta Ct values were converted to fold differences assuming amplification efficiency of 1.0. Adult expression level for each gene was set at 1.0.

Our *in silico* analyses also revealed that some clock genes were expressed at different levels between fetal and adult livers (Table S1). Since the *in silico* differences were sometimes below the 10-fold cut-off, we studied the actual differences further by real-time RT-PCR (Figure 3). *BMAL1*, *CLOCK*, *Cry1*, *Cry2* and *Per2* were expressed at levels ∼50% lower in the fetal liver, compared to adult liver. *Rev-erb α*, *Rev-erb β*, *Hlf* and *Tef* were expressed at much lower levels in the fetal liver. On the other hand, *Per1* was expressed at levels about 2-fold higher in the fetal liver. Knocking out *Timeless* leads to embryonic lethality [15]. *Timeless*, which is known to participate in cell cycle checkpoint functions [16], is enriched in the fetal liver both *in silico* and by semi-quantitative RT-PCR analysis (Figure 2). Some transcripts under clock control and rhythmically expressed in the adult liver, such as *Alas1*, *Cyp7a1*, *Dbp*, *Tef* and *Hlf*, were enriched in the adult liver *in silico* and the differences were also confirmed by semi-quantitative or real time RT-PCR (Figures 2 and 3). Those genes are not rhythmically expressed in the fetal liver according to our microarray time series analysis (results presented in the accompanying paper). They function in heme biosynthesis, bile acid production and xenobiotic detoxification [17], [18], [19], functions that are likely immature in the fetal liver but develop during the postnatal period. Electron transport and oxidative phosphorylation in the mitochondria play important roles in energy production and compartmentalized redox regulation [20], [21]. Mitochondrial energy production is known to be immature in the fetal liver, partly due to inefficient coupling of respiration with oxidative phosphorylation [22], [23]. We found the mitochondrial uncoupling protein *UCP2* [24], [25] was enriched in the fetal liver (Figure 2).

It should be noted that rhythmic transcripts in the adult liver were not always expressed at lower levels in the fetal liver. In fact, about half of the rhythmic transcripts in the adult liver were expressed at higher levels in the fetal liver (Figure S3A and S3B), similar to the general trend for all transcripts (Figures 1B and S1B).

In contrast to the adult liver, the fetal liver appears to lack circadian oscillation. Overall approximately 1000 transcripts are differentially enriched between fetal and adult livers. Whether or how clock oscillation contributes to those developmental differences in gene expression, however, is not clear. In a mouse model with a conditionally-active liver clock [26], the liver also developed without oscillating clocks (by repressing *BMAL1* expression), but the clock could be readily re-initiated by resuming *BMAL1* expression in the adult liver. Transcriptome comparison between those adult mouse livers with and without clocks [26] revealed an overall correlation of 0.95 (Figure S4). The relatively small divergence, which is considerably less than that seen between fetal and adult livers (Figures 1 and S1), might be taken as the contribution of clock oscillation to the overall transcriptome. Thus the transcriptome differences we observed between fetal and adult livers likely represent developmental differences largely independent of clock oscillation status.

## Discussion

Through transcriptome comparison, we identified genes whose relative expression levels diverged between fetal and adult mouse livers. The divergence was then used as the guide to infer differences in functional specializations. The results thus derived are in general agreement with known functional differences between fetal and adult livers. Of particular interest, our analysis reveals that clock genes and some clock-controlled genes are differentially expressed between fetal and adult mouse livers.

Multiple clock genes are expressed at different relative levels between fetal and adult livers. If mechanisms related to development are not taken into account, such differential expression patterns of clock genes within the same tissue are not readily explicable by the current clockwork model [2]. However, it is not without precedence. For example, clock gene expression in ES cells, when compared to NIH3T3 cells or during differentiaion, also show patterns not fully consistent with the clockwork model [6]. It has been reported that ES cells may lack endogenous circadian oscillation [6]. However, without imaging studies at the single cell level, a similar conclusion could not be readily drawn for fetal liver cells. Gene specific changes in clock gene expression have been observed in SIRT1 deficient cells, without abolishing cellular rhythmicity [27]. However, fetal liver lacks tissue level expression rhythms of clock genes and many clock-controlled genes, despite the fact that other rhythmic transcripts could still be identified in the fetal liver. It thus appears that mechanisms other than the canoniclal clockwork [28], [29], or clock genes that are dispensable for the adult liver clock [30], [31], might play roles in sustaining rhythmic gene expression in the fetal liver. In addition, systemic cues from the dam may also impose gene expression rhythms in the fetal liver. To test the latter possibilty, we compared the phases of rhythmic transcripts in the fetal liver with those in the adult mouse liver (Table S4). Multiple factors contribute to gene expression rhythms in the adult liver: the endogenous clock, systemic, neural and hormonal cues, and body temperature fluctuations [26], [32], [33], [34], [35], [36]. Those factors may have distinct, combinatorial, and even conflicting effects on liver gene expression and on the clockwork under specific feeding conditions [36], [37], [38], [39]. Currently, we do not know the nature of the maternal cues that gain access to the fetal liver through the placenta [40], [41]. Fetal and adult livers also have obvious differences in their transcriptomes that potentially could lead to their differential responses even to the same stimuli. Nevertheless, we found that phases of those rhythmic transcripts were positively correlated between our series 2 fetal liver data and the adult liver data (Table S4 and Figure S5A and S5B ). However, phases of rhythmic transcripts in our series 1 data were correlated less well with those of the adult liver, likely due to the possible presence of irregularities in maternal feeding in this group of fetal mice (discussed in the accompanying paper). Since clock genes and many known clock-controlled genes were found not to be rhythmically expressed in the fetal liver, the possibility that gene expression rhythms in the fetal liver resulted from maternal influence could not be excluded. An interesting aspect of this proposed mechanism is that it requires maternal influence to bypass the fetal core clock genes and engage output genes directly.

While the transcription-translation feedback loop model of the clockwork stresses the cell autonomous nature of circadian oscillation, it has also been suggested that the cellular clock is an interface bridging input and output pathways and the clockwork is intimately linked with cellular metabolism [42]. Thus the oscillation status of the clock, which we refer to as canonical clock gene expression, is also linked with its functional state: output control and responsiveness to input stimuli. Such interactions with cellular metabolism and entrainment cues [43], [44], [45], while evident in the adult liver, might be quite different in the fetal liver. In adult mouse liver, *Rev-erb α* plays an important role in linking the circadian clock to lipid and cholesterol metabolism [19], [46], [47], [48]. We found both *Rev-erb α* and *Alas1* (involved in synthesizing heme, the ligand of *Rev-erb α* [47]) were expressed at lower levels in the fetal liver. Another important link between the clockwork and metabolism is through SIRT1 [27], [49], whose activity is regulated by NAD+ level 50,51. Structural and functional maturation of mitochondrial energy production occur rapidly after birth, along with a dramatic increase in cellular redox ratio ([NAD+]/[NADH]) [52], [53]. The fetal liver is in a reducing environment that likely limits SIRT1 function, a situation having effects on clock gene expression [27]. Indeed, SIRT1 deficiency has been reported to suppress clock oscillation amplitude [27] (but see [49]). We also found that DNA methyltransferases *Dnmt1*, *Dnmt3a* and *Dnmt3b* were all expressed at higher relative levels in the fetal liver. Epigenetic profiles, such as DNA methylation and histone modifications [54], [55], are known to change during development [56], [57]. Such epigenetic changes, while remain to be investigated further, likely could account for some of the observed differences in gene expression, including the expression of clock genes and clock-controlled genes, between fetal and adult livers.

The possible lack of endogenous circadian oscillation in the fetal liver does not preclude non-clock functions of clock genes. Mouse embryonic fibroblasts (MEFs) bearing the *CLOCK^Δ19^* mutation are remarkably deficient in proliferation capacity in culture [58]. Although we found *CLOCK* was expressed at a lower relative level in the fetal liver, the fetal liver is known to have high proliferative potential [12]. Both positive and negative regulators of cell cycle are enriched in the fetal liver, likely reflecting the increased need for DNA repair during cellular proliferation, as DNA repair activities are intimately linked to cell cycle checkpoints [59]. Although reciprocal interactions between the circadian clock and the cell cycle machinery have been observed [60], [61], there are also cases in which they are possibly dissociated [62]. Expression levels of some clock genes are significantly down-regulated in the zygote following the fertilization event [63], [64]. Mouse ES cells, which are rapidly proliferating cells [65], also do not seem to possess oscillating circadian clocks [6]. The fetal mouse liver cells might also proliferate *in utero* without apparent oscillation of its cellular clocks.

The *in utero* to *in vitro* change was considered the key stimulus to set the phase of circadian oscillation at the tissue level for the fetal mouse liver [9]. It is unlikely that increased coherence in cellular rhythms due to maturation of cells could account for the rapid appearance of tissue level oscillation in culture [66]. Rather, *de novo* oscillation may have been initiated by the fetal explant culturing procedure. Potentially influencing molecular factors likely differ between environments of explant culture and *in utero*. The intrauterine mileau may provide conflicting signals which actively suppress tissue-level rhythmicity of the fetal liver. Explantation may remove these signals and provide a stimulus for cellular synchronization. Fetal tissues may be particularly susceptible to re-setting, owing to extremely low amplitude rhythmicity of clock genes – rhythmicity so low in amplitude that it was not detected in studies of fetal liver, including ours. Change in oxygen level represents another factor impacting on fetal tissues. The phase of clock oscillation in postnatal tissues appear to be relatively unaffected by placement into explant culture [67], [68]. Furthermore, exposure to higher oxygen level following natural birth or dissection of fetal liver out of the uterus can trigger maturation of mitochondrial energy production and lead to increase in cellular redox ratio [22], [23]. Future studies involving transcriptome comparisons between fetal liver tissues *in utero* and explants cultured *in vitro* might reveal the cellular effects caused by the environmental change.

Considering the lack of tissue level expression rhythms of clock genes and clock-controlled genes and the general differences between fetal and adult liver transcriptomes, the circadian clock in the fetal liver might not operate by established clockwork mechanisms for either intrinsic oscillation or interactions with cellular processes. There is also evidence that circadian oscillations are differentially started in peripheral tissues during postnatal development [68]. Daily profiles of clock gene expression were also found to change rather idiosyncratically during postnatal development in the rat liver before their canonical expression patterns were established [8]. Circadian rhythms, from cellular to organism levels, are of adaptive values to adult life. Maturation of clock mechanisms might be part of the developmental program of terminal differentiation to gain full-fledged cellular functions for adult life, at least in the livers of rodents such as rats and mice.

## Supporting Information

Figure S1 **Comparisons between fetal (series 1 data) and adult liver transcriptomes.** (**A**) Scatterplot of normalized average expression values in fetal (y-axis, series 1 data) and adult (x-axis) liver. Pair-wise values for 22626 probe sets were plotted. *r* = 0.70, *P*<0.01. (**B**) Fold difference in normalized expression values between fetal (series 1 data) and adult liver for 22626 probe sets. Ratios (fetal: adult) were plotted against their ranks.
(TIF)

Figure S2 **Comparisons between the two series of fetal liver transcriptome data.** (**A**) Scatterplot of GC-RMA normalized and scaled average expression values in series 1 (y-axis) and series 2 (x-axis) microarray data. (**B**) Pairwise fold difference in average expression values. Ratios (series 1: series 2, for all probe sets) were plotted against their ranks. The most dramatic probe set differences between the two series were within the 10-fold range, with the exception of 4 that fell below 15-fold.
(TIF)

Figure S3 **Differences in relative fetal and adult expression levels for rhythmic transcripts in the adult liver.** Comparisons were made between fetal and adult relative expression values for rhythmic transcripts in the adult mouse liver (*BH.Q.* <0.1 in GSE11923; 6478 probe sets) that were also represented in our microarray (4755 probe sets). Ratios (fetal: adult) were plotted against their ranks. (**A**) Series 1 *vs.* adult. (**B**) Series 2 *vs.* adult.
(TIF)

Figure S4 **Liver transcriptome comparisons between adult mice with and without the liver clock.** Scatterplot of pairwise average expression values (E-MEXP-842 [26] from ArrayExpress) for mice with and without doxycycline treatment were compared.
(JPG)

Figure S5 **Phase distribution of rhythmic transcripts in adult and fetal livers.** (A) Scatterplot of pairwise linear phase values for 619 rhythmic transcripts common to adult WT and fetal series 1 data. (linear phase corrrelation: −0.076; angular phase correlation: −0.025, *p*<0.05). (**B**) Scatterplot of pairwise linear phase values for 325 rhythmic transcripts common to adult WT and fetal series 2 data (linear phase correlation: 0.458; angular phase correlation: 0.095, *p*<0.05). For detailed information, see Table S4.
(TIF)

Table S1 **Comparisons between relative expression levels in fetal and adult WT livers.** Average daily expression values for 22626 common probe sets across platforms were scaled and pairwise comparisons were made between our fetal liver data series and the GSE11923 adult mouse liver data. Commonly enriched probe sets in series 1 and 2 were also identified.
(XLS)

Table S2 **Comparisons between expression values of adult mice obtained on two consecutive days.** Average expression values for the first and last 24 time points in GSE11923 were calculated for all probe sets and their ratios were calculated and ranked.
(XLS)

Table S3 **DAVID analysis results for transcripts enriched in either fetal or adult liver.** Probe sets that were differentially enriched in either fetal (906) or adult (460) liver transcriptomes were analyzed. Commonly enriched probe sets were determined by comparing our series 1 or series 2 fetal data against GSE11923. Enriched transcripts datasets were processed by DAVID to determine annotation clustering.
(XLS)

Table S4 **Phase correlation analysis between common rhythmic transcripts in fetal and adult livers.** Expression peaks were determined for rhythmic transcripts (*p*<0.1 in either of our two fetal data series, *BH.Q*.<0.1 in GSE11923. JTK_CYCLE analyses were performed using a fixed 24 hours period to ease derivation of peak phases) according to the Lag values given by JTK_CYCLE. Linear (circadian time of expression peak) and angular (peak time expressed as degrees) phase values were determined. Correlation analyses of phase distributions were performed using either linear or angular data. 619 common transcripts were found between adult and series 1 data (linear phase corrrelation: −0.076; angular phase correlation: −0.026, *p*<0.05). 325 common rhythmic transcripts were found between adult and series 2 data (linear phase correlation: 0.458; angular phase correlation: 0.095, *p*<0.05). Overall, 44 rhythmic transcripts were found common to adult and both fetal data series. Correlation analyses indicated that the phases of those 44 transcripts were better correlated between fetal series 2 and adult data than between fetal series 1 and adult data.
(XLS)

Table S5 **Primers for semi-quantitative and real-time RT-PCR with corresponding GenBank accession numbers.** All PCR amplicon products were verified by cloning and sequencing.
(XLS)

## References

[1] Ukai H, Ueda HR (2010). Systems biology of mammalian circadian clocks.. Annu Rev Physiol.

[2] Takahashi JS, Hong HK, Ko CH, McDearmon EL (2008). The genetics of mammalian circadian order and disorder: implications for physiology and disease.. Nat Rev Genet.

[3] Hattar S, Lucas RJ, Mrosovsky N, Thompson S, Douglas RH (2003). Melanopsin and rod-cone photoreceptive systems account for all major accessory visual functions in mice.. Nature.

[4] Panda S, Antoch MP, Miller BH, Su AI, Schook AB (2002). Coordinated transcription of key pathways in the mouse by the circadian clock.. Cell.

[5] Storch KF, Lipan O, Leykin I, Viswanathan N, Davis FC (2002). Extensive and divergent circadian gene expression in liver and heart.. Nature.

[6] Yagita K, Horie K, Koinuma S, Nakamura W, Yamanaka I (2010). Development of the circadian oscillator during differentiation of mouse embryonic stem cells in vitro.. Proc Natl Acad Sci U S A.

[7] Dolatshad H, Davis FC, Johnson MH (2009). Circadian clock genes in reproductive tissues and the developing conceptus.. Reprod Fertil Dev.

[8] Sladek M, Jindrakova Z, Bendova Z, Sumova A (2007). Postnatal ontogenesis of the circadian clock within the rat liver.. Am J Physiol Regul Integr Comp Physiol.

[9] Dolatshad H, Cary AJ, Davis FC (2010). Differential expression of the circadian clock in maternal and embryonic tissues of mice.. PLoS One.

[10] Bohme HJ, Sparmann G, Hofmann E (1983). Biochemistry of liver development in the perinatal period.. Experientia.

[11] Hughes ME, DiTacchio L, Hayes KR, Vollmers C, Pulivarthy S (2009). Harmonics of circadian gene transcription in mammals.. PLoS Genet.

[12] Gruppuso PA, Bienieki TC, Faris RA (1999). The relationship between differentiation and proliferation in late gestation fetal rat hepatocytes.. Pediatr Res.

[13] Girard J, Ferre P, Pegorier JP, Duee PH (1992). Adaptations of glucose and fatty acid metabolism during perinatal period and suckling-weaning transition.. Physiol Rev.

[14] da Rocha ST, Edwards CA, Ito M, Ogata T, Ferguson-Smith AC (2008). Genomic imprinting at the mammalian Dlk1-Dio3 domain.. Trends Genet.

[15] Gotter AL, Manganaro T, Weaver DR, Kolakowski LF, Possidente B (2000). A time-less function for mouse timeless.. Nat Neurosci.

[16] Unsal-Kacmaz K, Mullen TE, Kaufmann WK, Sancar A (2005). Coupling of human circadian and cell cycles by the timeless protein.. Mol Cell Biol.

[17] Kaasik K, Lee CC (2004). Reciprocal regulation of haem biosynthesis and the circadian clock in mammals.. Nature.

[18] Gachon F, Olela FF, Schaad O, Descombes P, Schibler U (2006). The circadian PAR-domain basic leucine zipper transcription factors DBP, TEF, and HLF modulate basal and inducible xenobiotic detoxification.. Cell Metab.

[19] Le Martelot G, Claudel T, Gatfield D, Schaad O, Kornmann B (2009). REV-ERBalpha participates in circadian SREBP signaling and bile acid homeostasis.. PLoS Biol.

[20] Houtkooper RH, Canto C, Wanders RJ, Auwerx J (2010). The secret life of NAD+: an old metabolite controlling new metabolic signaling pathways.. Endocr Rev.

[21] Koch-Nolte F, Fischer S, Haag F, Ziegler M (2011). Compartmentation of NAD+-dependent signalling.. FEBS Lett.

[22] Pollak JK (1975). The maturation of the inner membrane of foetal rat liver mitochondria.. Biochem J.

[23] Valcarce C, Navarrete RM, Encabo P, Loeches E, Satrustegui J (1988). Postnatal development of rat liver mitochondrial functions. The roles of protein synthesis and of adenine nucleotides.. J Biol Chem.

[24] Krauss S, Zhang CY, Lowell BB (2005). The mitochondrial uncoupling-protein homologues.. Nat Rev Mol Cell Biol.

[25] Lamia KA, Storch KF, Weitz CJ (2008). Physiological significance of a peripheral tissue circadian clock.. Proc Natl Acad Sci U S A.

[26] Kornmann B, Schaad O, Bujard H, Takahashi JS, Schibler U (2007). System-driven and oscillator-dependent circadian transcription in mice with a conditionally active liver clock.. PLoS Biol.

[27] Asher G, Gatfield D, Stratmann M, Reinke H, Dibner C (2008). SIRT1 regulates circadian clock gene expression through PER2 deacetylation.. Cell.

[28] Storch KF, Weitz CJ (2009). Daily rhythms of food-anticipatory behavioral activity do not require the known circadian clock.. Proc Natl Acad Sci U S A.

[29] O'Neill JS, Reddy AB (2011). Circadian clocks in human red blood cells.. Nature.

[30] DeBruyne JP, Weaver DR, Reppert SM (2007). Peripheral circadian oscillators require CLOCK.. Curr Biol.

[31] Debruyne JP, Noton E, Lambert CM, Maywood ES, Weaver DR (2006). A Clock Shock: Mouse CLOCK Is Not Required for Circadian Oscillator Function.. Neuron.

[32] Terazono H, Mutoh T, Yamaguchi S, Kobayashi M, Akiyama M (2003). Adrenergic regulation of clock gene expression in mouse liver.. Proc Natl Acad Sci U S A.

[33] Damiola F, Le Minh N, Preitner N, Kornmann B, Fleury-Olela F (2000). Restricted feeding uncouples circadian oscillators in peripheral tissues from the central pacemaker in the suprachiasmatic nucleus.. Genes Dev.

[34] Reinke H, Saini C, Fleury-Olela F, Dibner C, Benjamin IJ (2008). Differential display of DNA-binding proteins reveals heat-shock factor 1 as a circadian transcription factor.. Genes Dev.

[35] Buhr ED, Yoo SH, Takahashi JS (2010). Temperature as a universal resetting cue for mammalian circadian oscillators.. Science.

[36] Vollmers C, Gill S, DiTacchio L, Pulivarthy SR, Le HD (2009). Time of feeding and the intrinsic circadian clock drive rhythms in hepatic gene expression.. Proc Natl Acad Sci U S A.

[37] Le Minh N, Damiola F, Tronche F, Schutz G, Schibler U (2001). Glucocorticoid hormones inhibit food-induced phase-shifting of peripheral circadian oscillators.. Embo J.

[38] Guo H, Brewer JM, Lehman MN, Bittman EL (2006). Suprachiasmatic regulation of circadian rhythms of gene expression in hamster peripheral organs: effects of transplanting the pacemaker.. J Neurosci.

[39] Vujovic N, Davidson AJ, Menaker M (2008). Sympathetic input modulates, but does not determine, phase of peripheral circadian oscillators.. Am J Physiol Regul Integr Comp Physiol.

[40] Weaver DR, Reppert SM (1989). Periodic feeding of SCN-lesioned pregnant rats entrains the fetal biological clock.. Brain Res Dev Brain Res.

[41] Reppert SM, Schwartz WJ (1986). Maternal endocrine extirpations do not abolish maternal coordination of the fetal circadian clock.. Endocrinology.

[42] Lakin-Thomas PL (2006). Transcriptional feedback oscillators: maybe, maybe not.. J Biol Rhythms.

[43] Bass J, Takahashi JS (2010). Circadian integration of metabolism and energetics.. Science.

[44] Schibler U (2009). The 2008 Pittendrigh/Aschoff lecture: peripheral phase coordination in the mammalian circadian timing system.. J Biol Rhythms.

[45] Eckel-Mahan K, Sassone-Corsi P (2009). Metabolism control by the circadian clock and vice versa.. Nat Struct Mol Biol.

[46] Alenghat T, Meyers K, Mullican SE, Leitner K, Adeniji-Adele A (2008). Nuclear receptor corepressor and histone deacetylase 3 govern circadian metabolic physiology.. Nature.

[47] Yin L, Wu N, Curtin JC, Qatanani M, Szwergold NR (2007). Rev-erbalpha, a heme sensor that coordinates metabolic and circadian pathways.. Science.

[48] Feng D, Liu T, Sun Z, Bugge A, Mullican SE (2011). A circadian rhythm orchestrated by histone deacetylase 3 controls hepatic lipid metabolism.. Science.

[49] Nakahata Y, Kaluzova M, Grimaldi B, Sahar S, Hirayama J (2008). The NAD+-dependent deacetylase SIRT1 modulates CLOCK-mediated chromatin remodeling and circadian control.. Cell.

[50] Nakahata Y, Sahar S, Astarita G, Kaluzova M, Sassone-Corsi P (2009). Circadian control of the NAD+ salvage pathway by CLOCK-SIRT1.. Science.

[51] Ramsey KM, Yoshino J, Brace CS, Abrassart D, Kobayashi Y (2009). Circadian clock feedback cycle through NAMPT-mediated NAD+ biosynthesis.. Science.

[52] Philippidis H, Ballard FJ (1969). The development of gluconeogenesis in rat liver: experiments in vivo.. Biochem J.

[53] Ballard FJ (1971). The development of gluconeogenesis in rat liver. Controlling factors in the newborn.. Biochem J.

[54] Li E (2002). Chromatin modification and epigenetic reprogramming in mammalian development.. Nat Rev Genet.

[55] Cedar H, Bergman Y (2009). Linking DNA methylation and histone modification: patterns and paradigms.. Nat Rev Genet.

[56] Meissner A, Mikkelsen TS, Gu H, Wernig M, Hanna J (2008). Genome-scale DNA methylation maps of pluripotent and differentiated cells.. Nature.

[57] Reik W, Dean W, Walter J (2001). Epigenetic reprogramming in mammalian development.. Science.

[58] Miller BH, McDearmon EL, Panda S, Hayes KR, Zhang J (2007). Circadian and CLOCK-controlled regulation of the mouse transcriptome and cell proliferation.. Proc Natl Acad Sci U S A.

[59] Sancar A, Lindsey-Boltz LA, Unsal-Kacmaz K, Linn S (2004). Molecular mechanisms of mammalian DNA repair and the DNA damage checkpoints.. Annu Rev Biochem.

[60] Matsuo T, Yamaguchi S, Mitsui S, Emi A, Shimoda F (2003). Control mechanism of the circadian clock for timing of cell division in vivo.. Science.

[61] Nagoshi E, Saini C, Bauer C, Laroche T, Naef F (2004). Circadian gene expression in individual fibroblasts: cell-autonomous and self-sustained oscillators pass time to daughter cells.. Cell.

[62] Yeom M, Pendergast JS, Ohmiya Y, Yamazaki S (2010). Circadian-independent cell mitosis in immortalized fibroblasts.. Proc Natl Acad Sci U S A.

[63] Hamatani T, Carter MG, Sharov AA, Ko MS (2004). Dynamics of global gene expression changes during mouse preimplantation development.. Dev Cell.

[64] Amano T, Matsushita A, Hatanaka Y, Watanabe T, Oishi K (2009). Expression and functional analyses of circadian genes in mouse oocytes and preimplantation embryos: Cry1 is involved in the meiotic process independently of circadian clock regulation.. Biol Reprod.

[65] Aladjem MI, Spike BT, Rodewald LW, Hope TJ, Klemm M (1998). ES cells do not activate p53-dependent stress responses and undergo p53-independent apoptosis in response to DNA damage.. Curr Biol.

[66] O'Neill JS, Hastings MH (2008). Increased coherence of circadian rhythms in mature fibroblast cultures.. J Biol Rhythms.

[67] Yoshikawa T, Yamazaki S, Menaker M (2005). Effects of preparation time on phase of cultured tissues reveal complexity of circadian organization.. J Biol Rhythms.

[68] Yamazaki S, Yoshikawa T, Biscoe EW, Numano R, Gallaspy LM (2009). Ontogeny of circadian organization in the rat.. J Biol Rhythms.

