# Supplementary figures and images for: Transcriptome Comparison between Fetal and Adult Mouse Livers: Implications for Circadian Clock Mechanisms
Source: PLoS One. 2012 Feb 21;7(2):e31292. doi: 10.1371/journal.pone.0031292 (PMC3283632; doi:10.1371/journal.pone.0031292)

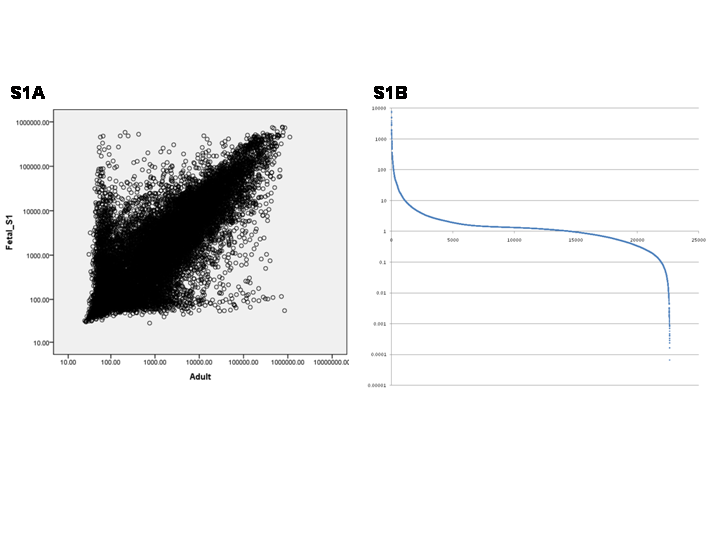

Supplement: Figure S1 — Comparisons between fetal (series 1 data) and adult liver transcriptomes. (A) Scatterplot of normalized average expression values in fetal (y-axis, series 1 data) and adult (x-axis) liver. Pair-wise values for 22626 probe sets were plotted. r = 0.70, P<0.01. (B) Fold difference in normalized expression values between fetal (series 1 data) and adult liver for 22626 probe sets. Ratios (fetal: adult) were plotted against their ranks. (TIF) [file pone.0031292.s001.tif]

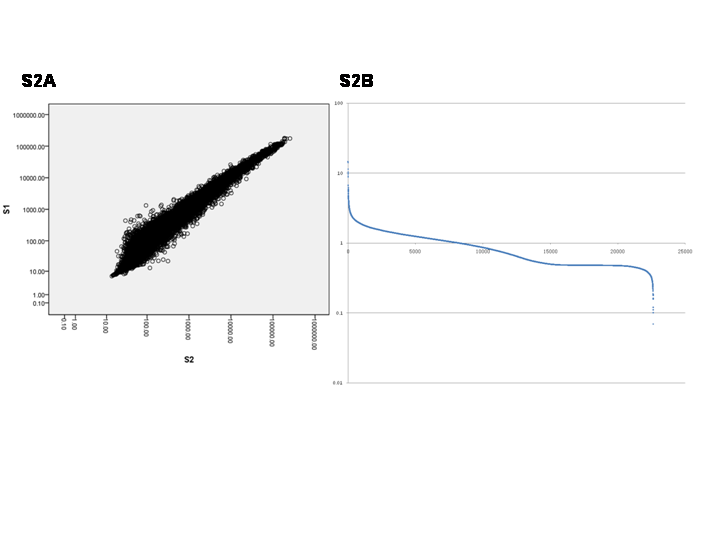

Supplement: Figure S2 — Comparisons between the two series of fetal liver transcriptome data. (A) Scatterplot of GC-RMA normalized and scaled average expression values in series 1 (y-axis) and series 2 (x-axis) microarray data. (B) Pairwise fold difference in average expression values. Ratios (series 1: series 2, for all probe sets) were plotted against their ranks. The most dramatic probe set differences between the two series were within the 10-fold range, with the exception of 4 that fell below 15-fold. (TIF) [file pone.0031292.s002.tif]

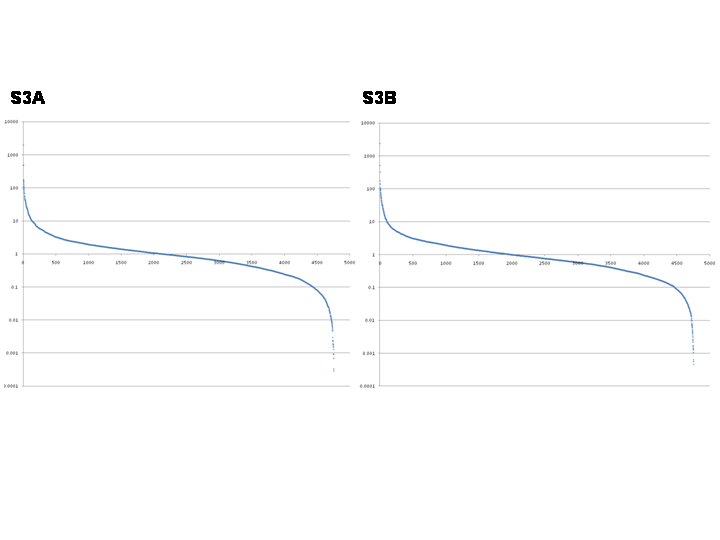

Supplement: Figure S3 — Differences in relative fetal and adult expression levels for rhythmic transcripts in the adult liver. Comparisons were made between fetal and adult relative expression values for rhythmic transcripts in the adult mouse liver (BH.Q. <0.1 in GSE11923; 6478 probe sets) that were also represented in our microarray (4755 probe sets). Ratios (fetal: adult) were plotted against their ranks. (A) Series 1 vs. adult. (B) Series 2 vs. adult. (TIF) [file pone.0031292.s003.tif]

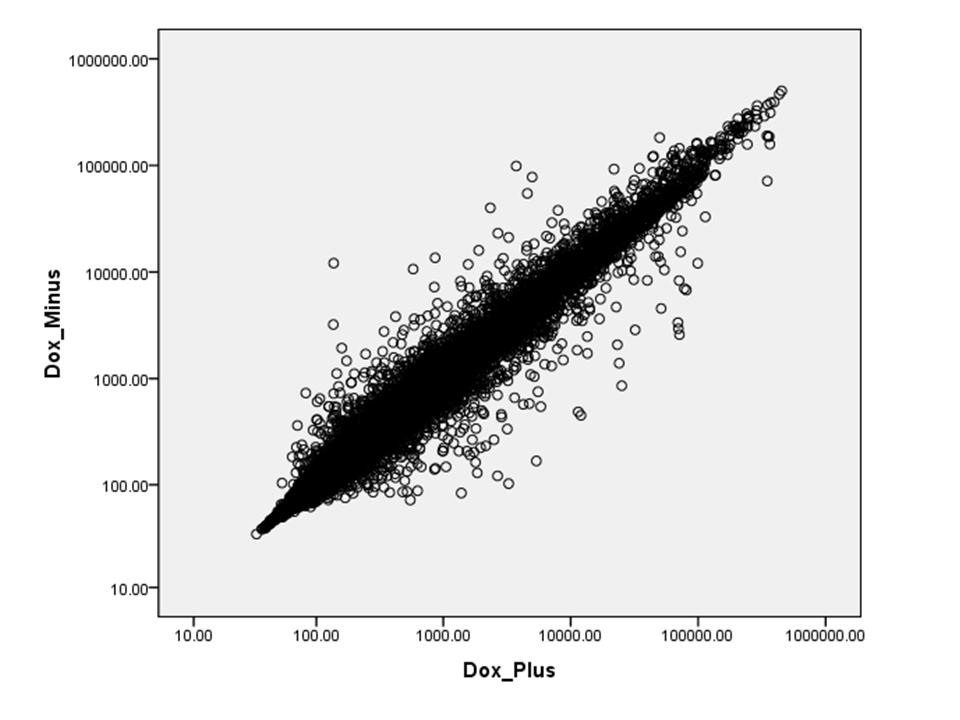

Supplement: Figure S4 — Liver transcriptome comparisons between adult mice with and without the liver clock. Scatterplot of pairwise average expression values (E-MEXP-842 [26] from ArrayExpress) for mice with and without doxycycline treatment were compared. (JPG) [file pone.0031292.s004.jpg]

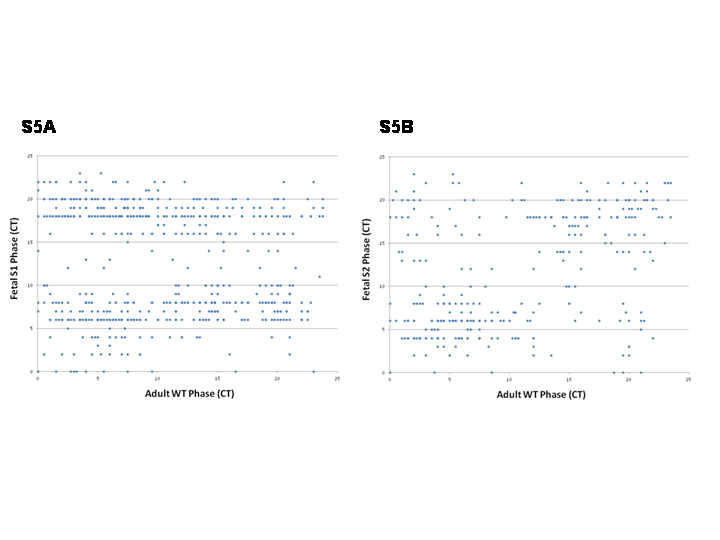

Supplement: Figure S5 — Phase distribution of rhythmic transcripts in adult and fetal livers. (A) Scatterplot of pairwise linear phase values for 619 rhythmic transcripts common to adult WT and fetal series 1 data. (linear phase corrrelation: −0.076; angular phase correlation: −0.025, p<0.05). (B) Scatterplot of pairwise linear phase values for 325 rhythmic transcripts common to adult WT and fetal series 2 data (linear phase correlation: 0.458; angular phase correlation: 0.095, p<0.05). For detailed information, see Table S4. (TIF) [file pone.0031292.s005.tif]
